# A 25-year retrospective, single center analysis of 343 WHO grade II/III glioma patients: implications for grading and temozolomide therapy

**DOI:** 10.1007/s00432-021-03511-y

**Published:** 2021-02-04

**Authors:** Eike Steidl, Katharina Filipski, Pia S. Zeiner, Marlies Wagner, Emmanouil Fokas, Marie-Therese Forster, Michael W. Ronellenfitsch, Iris Divé, Joachim P. Steinbach, Patrick N. Harter, Oliver Bähr

**Affiliations:** 1 Senckenberg Institute of Neurooncology, University Hospital, Goethe University Frankfurt am Main, Frankfurt am Main, Germany; 2 Institute of Neuroradiology, University Hospital, Goethe University Frankfurt am Main, Schleusenweg 2-16, 60528 Frankfurt am Main, Germany; 3 Department of Radiooncology, University Hospital, Goethe University Frankfurt am Main, Frankfurt am Main, Germany; 4 Frankfurt Cancer Institute (FCI), University Hospital, Goethe University Frankfurt am Main, Frankfurt am Main, Germany; 5 Department of Neurosurgery, University Hospital, Goethe University Frankfurt am Main, Frankfurt am Main, Germany; 6 Institute of Neurology (Edinger-Institute), University Hospital, Goethe University Frankfurt am Main, Frankfurt am Main, Germany; 7 German Cancer Consortium (DKTK), Partner site Frankfurt/ Mainz, Frankfurt am Main, Germany; 8 German Cancer Research Center (DKFZ), Heidelberg, Germany

**Keywords:** Low grade glioma, Astrocytoma, Oligodendroglioma, Grading, Treatment outcome, Temozolomide

## Abstract

**Purpose:**

Classification and treatment of WHO grade II/III gliomas have dramatically changed. Implementing molecular markers into the WHO classification raised discussions about the significance of grading and clinical trials showed overall survival (OS) benefits for combined radiochemotherapy. As molecularly stratified treatment data outside clinical trials are scarce, we conducted this retrospective study.

**Methods:**

We identified 343 patients (1995–2015) with newly diagnosed WHO grade II/III gliomas and analyzed molecular markers, patient characteristics, symptoms, histology, treatment, time to treatment failure (TTF) and OS.

**Results:**

IDH-status was available for all patients (259 mutant, 84 IDH1-R132H-non-mutant). Molecular subclassification was possible in 173 tumors, resulting in diagnosis of 80 astrocytomas and 93 oligodendrogliomas. WHO grading remained significant for OS in astrocytomas/IDH1-R132H-non-mutant gliomas (*p* < 0.01) but not for oligodendroglioma (*p* = 0.27). Chemotherapy (and temozolomide in particular) showed inferior OS compared to radiotherapy in astrocytomas (median 6.1/12.1 years; *p* = 0.03) and oligodendrogliomas (median 13.2/not reached (n.r.) years; *p* = 0.03). While radiochemotherapy improved TTF in oligodendroglioma (median radiochemotherapy n.r./chemotherapy 3.8/radiotherapy 7.3 years; *p* < 0.001/ = 0.06; OS data immature) the effect, mainly in combination with temozolomide, was weaker in astrocytomas (median radiochemotherapy 6.7/chemotherapy 2.3/radiotherapy 2.0 years; *p* < 0.001/ = 0.11) and did not translate to improved OS (median 8.4 years).

**Conclusion:**

This is one of the largest retrospective, real-life datasets reporting treatment and outcome in low-grade gliomas incorporating molecular markers. Current histologic grading features remain prognostic in astrocytomas while being insignificant in oligodendroglioma with interfering treatment effects. Chemotherapy (temozolomide) was less effective than radiotherapy in both astrocytomas and oligodendrogliomas while radiochemotherapy showed the highest TTF in oligodendrogliomas.

**Supplementary Information:**

The online version contains supplementary material available at 10.1007/s00432-021-03511-y.

## Introduction

Classification and treatment of WHO grade II and III gliomas have changed dramatically in the past decade. Approximately 80% of these tumors carry mutations of the isocitrate dehydrogenase (IDH) 1 or 2 genes, with 93% occurrence of the canonical IDH1-R132H mutation (Yan et al. 2009; Hartmann et al. 2009; Reuss et al. 2015a). Among IDH-mutant (IDH-mut) tumors, oligodendrogliomas are defined by an additional 1p/19q chromosomal codeletion. In addition, frequent TERT (telomerase reverse transcriptase) promoter mutations and a lack of nuclear trimethylation at lysine 27 of histone 3 (H3K27me3) can be observed, whereas astrocytomas have frequent mutations in the α-thalassaemia/mental retardation syndrome X-linked (ATRX) and TP53 genes (Yan et al. 2009; Reifenberger et al. 1994; Louis et al. 2016). Oligodendrogliomas have the better prognosis, while homozygous CDKN2A (cyclin-dependent kinase N2A) deletions portend a worse prognosis in IDH-mut astrocytomas.

IDH wildtype astrocytomas of WHO grade II/III have a poorer prognosis (Brat et al. 2015; Weller et al. 2015). Although these tumors commonly have molecular profiles featuring EGFR (epidermal growth factor receptor) amplification, PTEN (phosphatase and tensin homolog) loss and homozygous CDKN2A deletion as well as TERT promoter mutations which are hallmarks of glioblastoma, they do not fulfill the diagnostic criteria of glioblastoma and have a better prognosis (Brat et al. 2015; Weller et al. 2015; Pekmezci et al. 2017; Ceccarelli et al. 2016). The current view is that these tumors represent earlier stages of tumors on a trajectory towards glioblastoma. A recent in-depth molecular study concluded that tumor formation initiates 3 to 6 years before the stage of glioblastoma is reached (Körber et al. 2019).

When excluding IDH wildtype tumors from lower grade glioma cohorts, the relevance of histological WHO grading is questionable. Several studies reported a loss of the ability to stratify for overall survival (OS) (Reuss et al. 2015b; Cimino and Holland 2019; Yoda et al. 2019; Aoki et al. 2018; Olar et al. 2015), while others still demonstrated significance (Pekmezci et al. 2017; Shirahata et al. 2018; Appay et al. 2019). Simultaneously, an updated grading with the implementation of molecular markers has been proposed (Shirahata et al. 2018; Appay et al. 2019; Louis et al. 2018; Deimling et al. 2018; Brat et al. 2020).

Current treatment algorithms for patients with gliomas of WHO grade II are mainly based on the criteria and long-term outcome of the RTOG 9802 trial (Buckner et al. 2016). In low-risk patients (i.e., patients up to 40 years of age who received a gross-total resection) adjuvant treatment can be deferred. In all other patients, adjuvant radiochemotherapy (RCT) with RT and a combination of lomustine, procarbazine and vincristine (PCV) represents the standard of care (Nabors et al. 2017). Extrapolating the results from two trials in WHO grade III oligodendroglioma (Bent et al. 2013; Cairncross et al. 2013) further strengthens this recommendation for WHO grade II oligodendroglioma, while the positive second interim analysis of the CATNON trial (Bent et al. 2017,2019) supports this strategy for IDH-mut grade II astrocytomas. Frequently, temozolomide is substituted for PCV, but the evidence for equal efficacy is insufficient. This is especially important since TMZ therapy might increase the mutational load of gliomas, with possible implications for subsequent therapies (Touat et al. 2020). Upfront chemotherapy strategies have been suggested primarily out of concern for RT associated neurotoxicity in long-term surviving patients (Wick et al. 2016). This may be most justified in patients with large tumor volumes or gliomatosis cerebri growth pattern (Glas et al. 2011).

In gliomas of WHO grade III, adjuvant RCT with PCV (for oligodendrogliomas) or temozolomide (for astrocytomas) is generally recommended. Of note, the efficacy of RCT in the subgroup of IDH wildtype grade II and III astrocytomas is in doubt (Bent et al. 2019).

Due to the long OS of patients with IDH-mut glioma, comprehensive data in molecularly defined cohorts outside clinical trials are rare. Hence, we conducted this retrospective, single center analysis to report real-life data on patient characteristics, OS in the light of an ongoing discussion about tumor grading and treatment outcomes with special regard to TMZ.

## Patients and methods

### Study design and patient population

This retrospective study was approved by the ethics committee of the University Hospital Frankfurt (reference number 4/09 SNO-10–2016).

We screened our electronic medical records for patients with WHO grade II/III gliomas diagnosed between 1995 and 2015. Overall, we identified 424 patients with histologically confirmed WHO grade II/III glioma (figure S1). For these patients we set up a database and documented patient characteristics, histology, molecular markers, treatment and outcome.

### Additional molecular tests

Employing a cost effective algorithm (Filipski et al. 2019) with little requirements for tissue, we aimed at the best possible molecular characterization of this cohort. We performed automated immunohistochemistry (IHC) on 4 µm thick slides of formalin-fixed and paraffin embedded tissue (Leica Bond III device, standard protocols with IDH1-R132H specific, ATRX specific and H3K27me3 specific antibodies). Based on the WHO 2016 classification we classified the tumors into three molecular groups: (1) IDH1-R132H-non-mutant (IDH1-R132H-nm) gliomas were discriminated from gliomas carrying an IDH mutation assessed by immunohistochemistry, IDH1/2 mutations in sequencing or that clustered with IDH-mutant gliomas through DNA methylation analysis (referred to as IDH-mut in the following). Tumors in the IDH1-R132H-nm cohort were furthermore reviewed for histological hallmarks of diffuse glioma to exclude a contamination with other entities that typically do not harbor an IDH mutation such as pilocytic astrocytoma, pleomorphic xanthoastrocytomas or gangliogliomas. IDH-mut gliomas were again separated into astrocytomas and oligodendrogliomas. According to current suggestions (Louis et al. 2018) and the immunohistochemistry-based algorithm by (Filipski et al. 2019; Feller et al. 2020) IDH-mut tumors were identified as (2) astrocytoma if 1p/19q was intact or ATRX was lost and nuclear H3K27me3 was retained. Tumors were classified as (3) oligodendroglioma if 1p/19q was codeleted or ATRX was retained and nuclear H3K27me3 was lacking.

### Outcome measures

For treatment outcomes OS was calculated from the start of the specific therapy to death from any cause. For all other analysis OS was defined as time from diagnosis (date of biopsy/resection) to death from any cause. Additionally, we introduced time to treatment failure (TTF) as a composite endpoint. TTF was defined as the interval from the beginning of one therapy to the beginning of any following therapy or death from any cause. Patients who did not reach an endpoint were censored with date of last contact.

### Statistics

We employed SPSS Statistics 26.0 (IBM Corp., Armonk, NY, USA). *p* < 0.05 was considered statistically significant. Survival analyses were performed using the Kaplan–Meier method. We used the single comparison log-rank test in case of proportional hazard distribution and otherwise the generalized Wilcoxon test to compare outcomes. Other intergroup differences were analyzed using the Mann–Whitney-*U*-test. Correlations were analyzed by Pearson coefficient *r*. The rates for primary symptoms were calculated considering only patients with available data.

## Results

### Patient characteristics and molecular subgroups

Patient characteristics and molecular markers are shown in Table 1. Molecular data could be obtained for 343 tumors. Tissue samples were unavailable or test results were inconclusive in the remaining 81 cases and consequently these patients were excluded from outcome analysis (Fig. S1). A total of 259 IDH-mut tumors were identified, from which 80 tumors could be further specified as IDH-mut astrocytoma (median follow-up 6.6 years) and 93 tumors were identified as IDH-mut oligodendroglioma (median follow-up 7.5 years). In 84 tumors no IDH1-R132H mutation was detected (median follow-up 2.3 years). The reclassification of tumors from the morphological WHO 2007 to the molecular WHO 2016 classification is shown in figure S2, including further information on presenting symptoms, spatial distribution and treatments.

**Table 1:** Patient characteristics

General characteristics	*n*
Total patient number	343
Age at diagnosis	
Median (range)	42 (19–82)
≥ 40	205 (59.8%)
Sex	
Female	172 (50.1%)
Presenting symptom known	294
Epileptic seizure	180 (61.2%)
Headache	28 (9.5%)
Neurologic deficit	27 (9.2%)
Incidental finding	35 (11.9%)
Other	35 (11.9%)
Molecular markers/classification (adapted from WHO 2016)	
IDH-mutant	259 (75.5%)
Astrocytoma	80 (23.3%)
DNA methylation analysis	38 (11.1%)
IHC/FISH/CISH/Sequencing	42 (12.2%)
Oligodendroglioma	93 (27.1%)
DNA methylation analysis	45 (13.1%)
IHC/FISH/CISH/Sequencing	48 (14%)
IDH1-R132H-nm	84 (24.5%)
DNA methylation analysis (IDH wildtype)	10 (2.9%)
IHC	74 (21.6%)

*IDH*, isocitrate-dehydrogenase; *IHC*, immunohistochemistry; *FISH*, fluorescent in situ hybridization; *CISH*, chromogenic in situ hybridization; *IDH1-R132H-nm*, IDH1-R132H-non-mutant; two presenting symptoms were present in 10 patients

### OS based on molecular diagnosis in relation to WHO grading

**Fig. 1:**
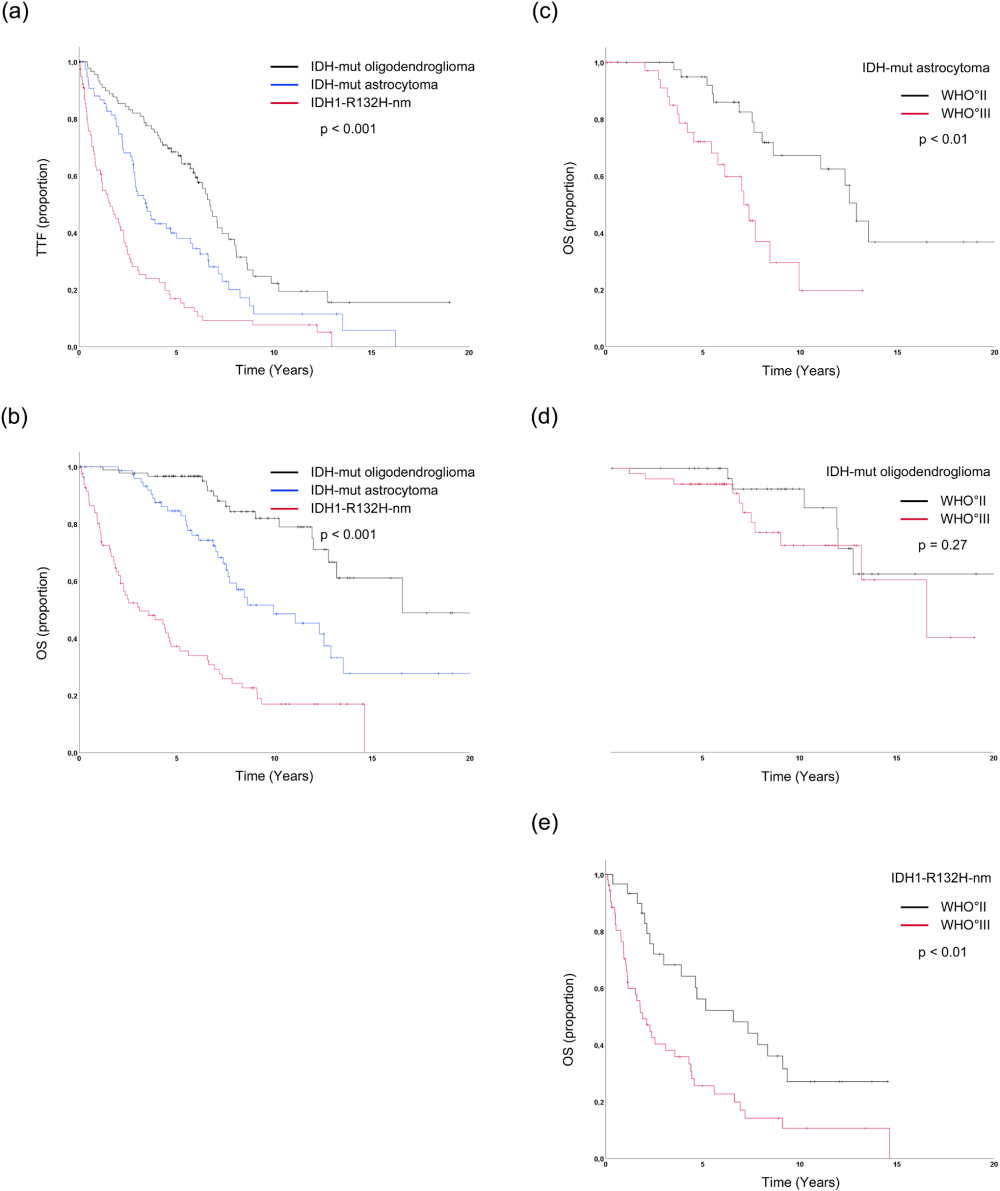
Outcome based on molecular classification and WHO grading. Kaplan–Meier curves for time to treatment failure (TTF, **a**) and overall survival (OS, **b**) of IDH-mutant (IDH-mut) oligodendroglioma (black), IDH-mut astrocytoma (blue) and IDH1-R132H-non-mutant (IDH1-R132H-nm) glioma (red). All differences are significant (*p* < 0.001, log-ranked test). **c–e** Show Kaplan–Meier curves for overall survival (OS) for WHO grade II/III IDH-mut astrocytoma (**c**), WHO grade II/III IDH-mut oligodendroglioma (**d**) and for IDH1-R132H-nm glioma (**e**). Differences between WHO grade II/III are significant (*p* < 0.05, log-ranked test) for IDH-mut astrocytoma and IDH1-R132H-nm tumors

IDH-mut oligodendrogliomas had the longest median OS (16.6 years), followed by IDH-mut astrocytomas (10.0 years) and IDH1-R132H-nm gliomas (3.1 years; *p* < 0.001 for all; Fig. 1a, b). OS analysis after separating each molecular subgroup by grade is shown in Fig. 1c–e. There were highly significant differences between grade II and III IDH-mut astrocytomas [*n* = 43/37; median OS (95%-confidence interval (CI)) 12.9 (11.9–13.9)/7.1 (5.5–8.7) years; *p* < 0.01] and between grade II and III IDH1-R132H-nm gliomas (*n* = 31/53; median OS (95% CI) 6.6 (2.4–10.8)/1.9 (1.0–2.8) years; *p* < 0.01). No significant difference was seen between grade II and III IDH-mut oligodendrogliomas (n = 38/55; median OS (95% CI) not reached (n.r.)/16.6 (10.5–22.6) years; *p* = 0.27). Concerning the patients characteristics and treatments there were no major differences between WHO grade II and III subgroups in median age (astrocytoma 37/37 years; IDH1-R132H-nm 50/55 years; oligodendroglioma 44/48 years) but grade III tumors were treated more frequently with RT or RCT during first-line therapy (astrocytoma 21/70%; IDH1-R132H-nm 32/74%; oligodendroglioma 24/60%). The proportion of patients who had received a resection was slightly higher for WHO grade II tumors than for grade III tumors in IDH-mut astrocytoma (91/86%) and notably higher in IDH1-R132H-nm tumors (71/53%), while it was lower in IDH-mut oligodendroglioma (74/94%). The corresponding curves for morphologically classified tumors are shown in Fig. S3.

### TTF and OS for molecular subgroups and non-surgical treatment

Treatment through first-, second- and third-line therapy is shown in Fig. S4. For all IDH-mut gliomas TTF decreased with every line of therapy (median 5.1/2.6/1.1 years respectively; Fig. S5). TTF correlated with OS in uncensored patients (*r* = 0.7–0.78; *p* < 0.001).

**Fig. 2:**
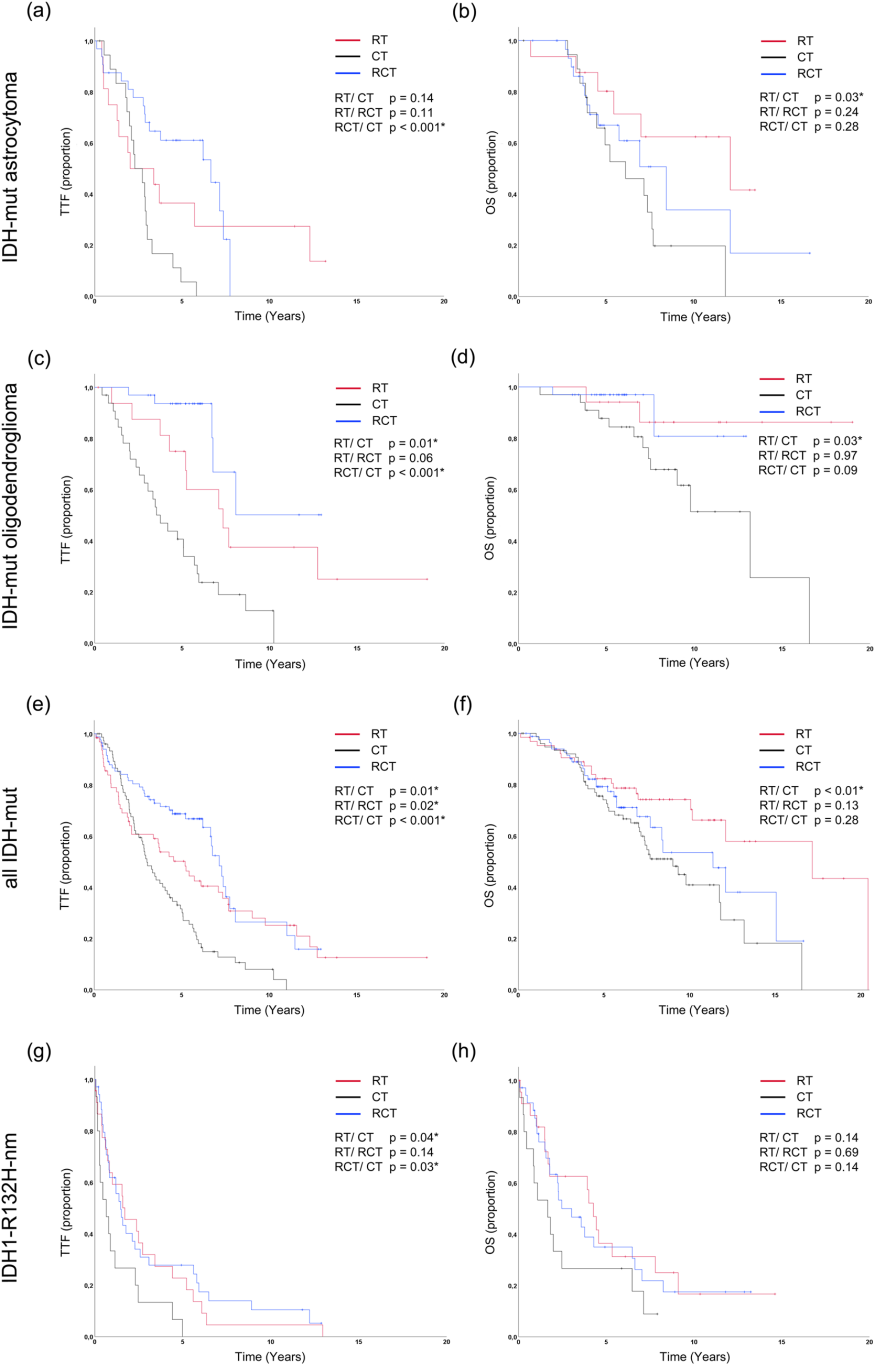
TTF and OS for molecular subgroups. Kaplan–Meier curves for time to treatment failure (TTF) and overall survival (OS) depending on first-line therapies (RT = radiotherapy, CT = chemotherapy, RCT = radiochemotherapy) for IDH-mutant (IDH-mut) astrocytoma (**a** and **b**), IDH-mut oligodendroglioma (**c** and **d**), all IDH-mut (**e** and **f**) and IDH1-R132H-non-mutant glioma (IDH1-R132H-nm (**g** and **h**). *p* values are shown for single comparison log-ranked tests or generalized Wilcoxon tests for the comparison of RCT and RT in **a** and **e**. A *indicates statistical significance (*p* < 0.05)

**Fig. 3:**
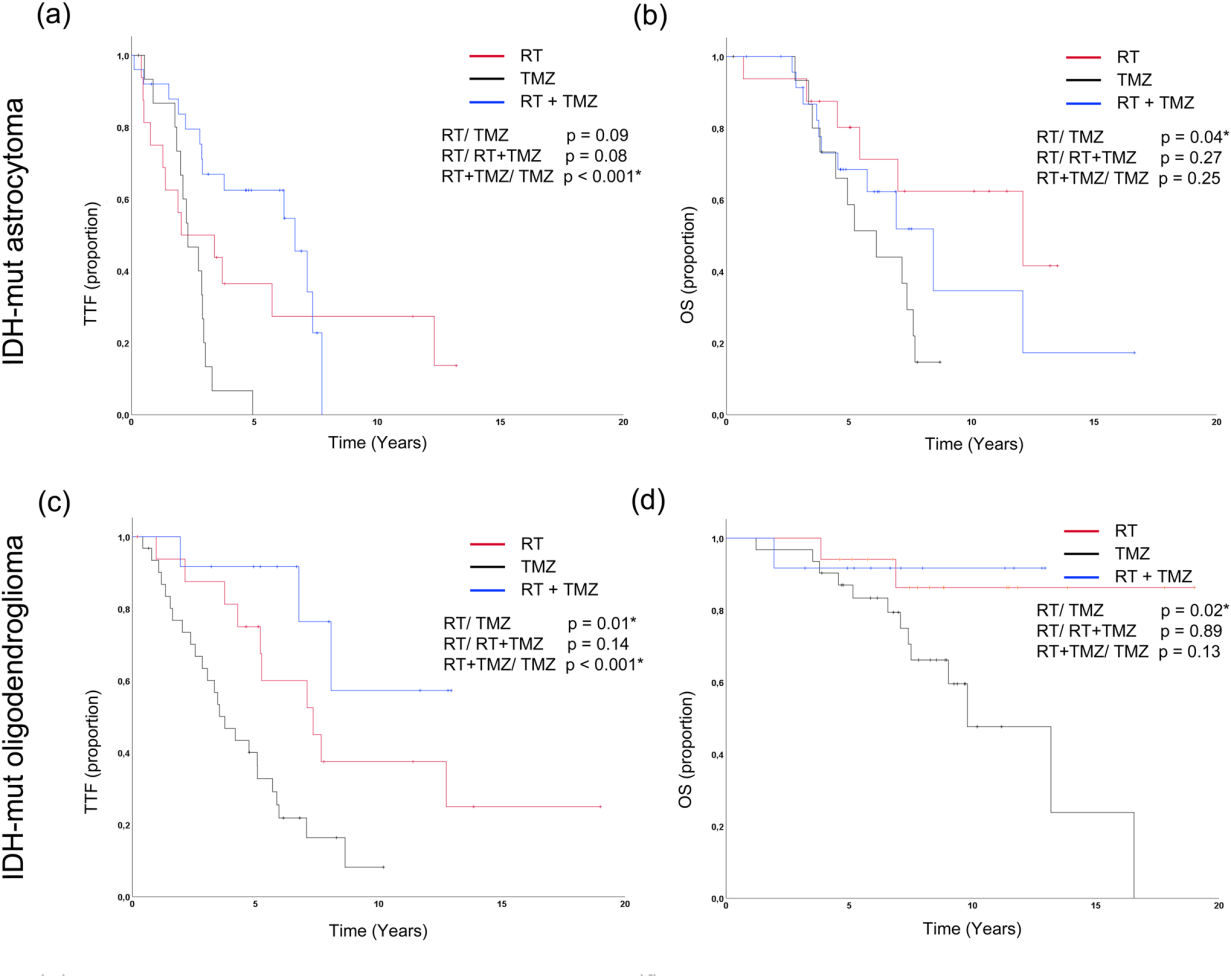
TTF and OS for molecular subgroups treated with temozolomide. Kaplan–Meier curves for time to treatment failure (TTF) and overall survival (OS) depending on first-line therapies for IDH-mutant (IDH-mut) astrocytoma (**a** and **b**) and IDH-mut oligodendroglioma (**c** and **d**). *p* values are shown for single comparison log-ranked tests. A * indicates statistical significance (*p* < 0.05). *RT* radiotherapy, *TMZ* temozolomide

For the molecular subgroups, the outcome of the first non-surgical therapy is shown in Fig. 2 and the characteristics are summarized in Table 2. In IDH-mut astrocytomas, TTF for RCT was superior to CT alone (median 6.7/2.3 years; *p* < 0.001) but not to RT (median 6.7/2.0 years; *p* = 0.11; Fig. 2a). OS was significantly higher for RT than for CT (median 12.1/6.1 years; *p* < 0.03) while RCT showed intermediate results (Fig. 2b). This effect persisted when specifically evaluating TMZ (*p* = 0.04; Fig. 3a, b). Interestingly, combined RT/TMZ was not superior to RT alone (*p* = 0.27). Subgroup characteristics showed higher rates for both WHO grade III tumors (64%/56%) and concurrent resections (72%/63%) as well as a slightly higher median age (38/34 years) in the RT/TMZ group compared to the RT group (Table S1).

**Table 2:** Characteristics for molecular subgroups shown in Fig. 2

Characteristics	Astro	Oligo	All IDH-mut	IDH1-R132H-nm
Radiotherapy				
* n*	16	17	64	23
Median age (years)	34	46	42	53
WHO grade II/III (%)	44/56	47/53	42/38	48/52
Resection (%)	63	65	45	35
Temozolomide (%)	0	0	0	0
Median TTF 95%-CI (years)	2.0 0.0–4.9	7.3 3.7–11.0	5.2 3.2–7.2	1.7 0.1–3.3
Median survival 95%-CI (years)	12.1 2.8–21.4	n.r	17.2 7.1–27.2	4.3 3.5–5.0
Chemotherapy				
* n*	19	33	77	15
Median age (years)	37	43	42	61
WHO grade II/III (%)	68/32	39/61	55/45	47/53
Resection (%)	47	64	48	13
Temozolomide (%)	84	94	86	80
Median TTF 95%-CI (years)	2.3 1.3–3.3	3.8 2.6–4.9	3.0 2.4–3.7	0.7 0.0–1.3
Median survival 95%-CI (years)	6.1 3.4–8.9	13.2 8.8.-17.5	9.0 6.9–11.2	1.7 0.5–2.8
Radiochemotherapy				
* n*	32	33	84	35
Median age (years)	38	42	40	52
WHO grade II/III (%)	47/53	27/73	31/69	20/80
Resection (%)	72	64	63	51
Temozolomide (%)	78	36	63	97
Median TTF 95%-CI (years)	6.7 2.6–10.7	n.r	7.1 6.4–7.9	1.5 1.0–2.0
Median survival 95%-CI (years)	8.4 5.3–11.6	n.r	11.4 7.9–14.8	3.0 1.4–4.7

*TTF*, time to treatment failure; *95%-CI*, 95% confidence interval; n.r., not reached; Astro., IDH-mut astrocytoma; Oligo., IDH-mut oligodendroglioma; all IDH-mut, all Isocitrate-Dehydrogenase mutant tumors including all IDH-mut astrocytoma, all IDH-mut oligodendroglioma, and tumors in that an IDH mutation was present but further molecular data was inconclusive or not available; IDH1-R132H-nm, IDH1-R132H-non-mutant; Chemotherapies without temozolomide were usually CCNU based regimens

In IDH-mut oligodendrogliomas the longest TTF was observed for RCT (median CT/RT/RCT 3.8/7.3/n.r.) years; RCT versus RT *p* = 0.06/versus CT *p* < 0.001; RT versus CT *p* = 0.01; Fig. 2c). RT remained superior to CT when analyzing OS (median n.r./13.2 years; *p* = 0.03; Fig. 2d). Noteworthy, a relevant number of IDH-mut oligodendroglioma RCT cases derived from more recent years, rendering the data less mature. Specific analysis of TMZ versus RT again confirmed superiority of RT for both TTF and OS (*p* = 0.01/0.02; Fig. 3c, d).

For all IDH-mut tumors, no significant OS signal for RT in comparison to RCT could be obtained. However, TTF and OS were significantly longer for RT as opposed to CT (*p* = 0.01; *p* < 0.01; Fig. 2e, f). In IDH1-R132H-nm tumors the inferior TTF for CT to both RT and RCT (*p* = 0.04/0.03) did not translate to significant OS differences with generally unfavorable OS (Fig. 2g, h).

## Discussion

Since long-term follow-up data in molecularly-defined lower grade glioma cohorts outside clinical trials are rare, we aimed to provide a comprehensive analysis of treatments and outcomes in this large single-center cohort, and to assess the prognostic value of tumor grading in a real-world setting.

General characteristics of our cohort and all subgroups such as age and sex distribution, predominant frontal lobe affection or seizures as prevalent presenting symptom were as to be expected from previous collectives (Table 1/2, Fig. S2) (Miller et al. 2019; Yeboa et al. 2019; Rasmussen et al. 2017; Lassman et al. 2011).

In line with previous studies (Reuss et al. 2015b; Cimino and Holland 2019; Aoki et al. 2018; Olar et al. 2015) our results confirm the missing significance of WHO grading for the prediction of OS in IDH-mut oligodendroglioma (Fig. 2d). In contrast to the cited studies (Brat et al. 2020), our simultaneous analysis of the different treatments enabled us to evaluate potential interferences. As a noteworthy finding, WHO grade III IDH-mut oligodendroglioma had been more frequently treated with RT or RCT as first-line therapy than WHO grade II IDH-mut oligodendroglioma (60%/24%, Table 2). Since these therapies might be more effective than CT alone based on our own data (Fig. 2) and prospective trials (Buckner et al. 2016; Cairncross et al. 2013; Wick et al. 2016), the outcomes for WHO grade III and II tumors could have been leveled through differing treatments. As preferences for different treatments vary from center to center (Yeboa et al. 2019; Ruff et al. 2019), often depending on WHO grading, this might also partly explain the contradictory findings for the impact of WHO grading reported in the literature (Pekmezci et al. 2017; Shirahata et al. 2018; Appay et al. 2019). In contrast, WHO grading remained significant for IDH-mut astrocytoma (Fig. 1c), contradicting several previous reports (Reuss et al. 2015b; Cimino and Holland 2019; Aoki et al. 2018; Olar et al. 2015). This finding is substantiated by the fact that WHO grade III tumors had again received more intense primary therapy (first-line RT or RCT 70% vs. 21% for WHO grade II) (Buckner et al. 2016; Bent et al. 2019; Baumert et al. 2016). Consequently, the OS differences in the natural course of the disease may be even higher. Interestingly, WHO grading also showed significance for IDH1-R132H-nm gliomas, which is in line with a previous publication (Aoki et al. 2018). The median OS of these patients (6.6 years and 1.9 years) was furthermore distinctly higher than the median OS reported in a population-based glioblastoma cohort (11.5 month) (Gramatzki et al. 2016), which might reflect a recently delineated trajectory of molecular evolution of these tumors (Körber et al. 2019) (Fig. 1e). In the light of promising results for molecular grading features like CDKN2A/B (Appay et al. 2019) a more profound combination of molecular and histologic features could be considered as a basis for the proposed renewal of the grading system (Deimling et al. 2018; Brat et al. 2020; Louis et al. 2017).

Regarding treatment outcomes for molecular oligodendroglioma, we were able to confirm the positive impact of RCT an TTF (Fig. 2c) as prospectively demonstrated by the RTOG 9402 and the EORTC 26,951 trials (Bent et al. 2013; Cairncross et al. 2013). The translation to OS was not yet apparent, probably due to the immaturity of the data in this subgroup. When comparing CT and RT only treatments, the NOA-4 trial (Wick et al. 2016) showed a higher TTF for RT that did not translate to significant OS differences. The interim analysis of the EORTC 22,033–26,033 trail (Baumert et al. 2016) did not show an improved PFS with OS results pending. Unlike these results, the TTF and OS difference between the CT or specifically TMZ and RT groups was significant in our study. This is in line with findings from the small cohort from the original design of the CODEL study (Jaeckle et al. 2016) which revealed inferiority of TMZ to RT and RT plus TMZ. Since the NOA-4 trial only enrolled WHO grade III tumors, the cohort studied in the CODEL trail more closely resembles our population. As there were no major imbalances concerning patient characteristics (Tables 2/A1) our findings strengthen the interpretation that TMZ monotherapy is not favorable in oligodendroglioma. Based on our data, the comparison of RT plus TMZ to RT plus PCV was not yet feasible. Further insight on this aspect might be offered by the results of the revised CODEL trial.

For IDH-mut astrocytoma, we found that RCT did not improve OS and that TMZ alone was associated with inferior OS compared to RT alone (Figs. 2/3b). This appears to be at odds with data from the CATNON/RTOG 9802 and NOA-04 trials (Buckner et al. 2016; Bent et al. 2019; Wick et al. 2016), demonstrating (i) improved OS following RCT and (ii) no difference in OS in patients receiving TMZ versus RT as first-line therapy. Concerning the first finding, there were no major imbalances between our RT and RCT subgroups regarding the rates of WHO grade III tumors, biopsy only patients or age (Table 2/A1). The predominant use of TMZ in our center is a noteworthy difference to the RTOG trail that investigated PCV. Nevertheless, our analysis focusing on TMZ (Fig. 3) involves treatment regimens comparable to the ones employed in the CATNON trail. Importantly, in contrast to our analysis the CATNON trail enrolled WHO grade III tumors only. While our median follow-up in IDH-mut astrocytomas was longer than the current follow-up of the CATNON trial (6.6/4.7 years), WHO grade II IDH-mut astrocytomas in our cohort also had a significantly longer median OS than WHO grade III tumors (5.8 years difference). As the OS difference in the RTOG 9802 trial (recruiting “high-risk” WHO grade II patients) only became evident after more than 7 years, it is well possible that our follow-up was still too short to reveal an OS benefit in the WHO grade II subgroup, distorting the overall result. On the other hand, there is no prospective evidence for the benefit of RCT in WHO grade II IDH-mut astrocytoma, since the analysis of the RTOG 9802 trial included all IDH-mut tumors without further subclassification. Therefore, our finding emphasizes the need for further studies on this specific subgroup. Considering the comparison of CT and RT alone (Fig. 2a, b), there were only two studies (Wick et al. 2016; Baumert et al. 2016) investigating outcomes for CT monotherapy in molecularly defined cohorts. OS data is only published for the NOA-4 trial (Wick et al. 2016), that exclusively enrolled WHO grade III tumors. As opposed to our analysis, the OS for TMZ was only numerically inferior to RT. With a much lower proportion of WHO grade III tumors, our CT/TMZ subgroups might have even been at an advantage concerning the patient characteristics compared to the RT subgroup (Tables 3/A1). Hence, our results imply that TMZ is inferior to RT in WHO grade II/III patients.

Lastly, the missing OS differences between treatment modalities in IDH1-R132-nm tumors is in line with findings in the respective subcohorts of the EORTC 22,033 (RT vs. TMZ) and the CATNON trail (RT + TMZ vs. RT) (Bent et al. 2019; Baumert et al. 2016). This might indicate a biological difference between IDH wildtype WHO grade II/III and WHO grade IV tumors, in which an OS benefit for RCT has been demonstrated (Stupp et al. 2005).

### Limitations

Major limitations of our study are the retrospective and monocentric nature as well as the limited power of the OS data for oligodendroglioma. Even though our patient cohort and the analyzed timespan were comparatively large, the median follow-up only reached 7.5 years. With regard to the histologic grading, it is noteworthy that tumor heterogeneity poses the risk of a sampling bias especially in cases with biopsy only. As this risk is common to most studies, differing findings might partially be attributable to this technical limitation. Concerning the molecular classification, screening all samples using sequencing or DNA methylation analysis was impossible, hence potentially disregarding rare IDH mutations [7–8% of all IDH-mut tumors (Hartmann et al. 2009)]. Furthermore, one can debate the use of TTF instead of PFS. The diagnosis of progression is usually based on MRI scans. To pinpoint the exact timepoint of progression is challenging in slowly but steadily growing glioma. Our composite TTF endpoint reflects the clinical significance of the underlying progression, even though it might not consider patients who deliberately avoided second-line therapy. The correlation to OS further supports TTF as a valuable marker.

## Conclusion

We present retrospective data on a large, single center, real-life cohort of WHO grade II/III glioma patients. Our study indicates that the current WHO grading system is still of relevance in molecularly classified IDH-mut astrocytoma. The absence of a prognostic effect of grading in IDH-mut oligodendroglioma may be partly due to effects of more intense treatment in grade III tumors that have not been considered in previous studies. Consequently, histologic grading parameters should still be taken into account for the upcoming renewal of the grading system.

Concerning treatment outcomes, our data suggests inferiority of TMZ to RT in all IDH-mut glioma. In IDH-mut astrocytomas we also did not find a significant positive impact of RT plus TMZ, possibly facilitated by the inclusion of WHO grade II tumors which might require extended follow-up. For IDH-mut oligodendroglioma, our study supports the finding of improved TTF for RCT. Regarding the choice of alkylating protocol, results of the CODEL trial comparing RT/PCV versus RT/TMZ are awaited.

## Supplementary Information

Below is the link to the electronic supplementary material.

Supplementary file1 (EPS 13413 KB)

Supplementary file2 (EPS 311924 KB)

Supplementary file3 (EPS 78589 KB)

Supplementary file4 (EPS 180500 KB)

Supplementary file5 (EPS 27823 KB)

Supplementary file6 (DOCX 19 KB)

Supplementary file7 (DOCX 23 KB)

## Data Availability

The datasets analyzed during the current study are not publicly available due to patient confidentiality but are available from the corresponding author on reasonable request.
