# Supplementary material for: A 25-year retrospective, single center analysis of 343 WHO grade II/III glioma patients: implications for grading and temozolomide therapy
Source: J Cancer Res Clin Oncol. 2021 Feb 4;147(8):2373–83. doi: 10.1007/s00432-021-03511-y (PMC8236451; doi:10.1007/s00432-021-03511-y)
Supplement: Supplementary file 6 — Supplementary file6 (DOCX 19 KB) [file 432_2021_3511_MOESM6_ESM.docx]

**Tab.S1** Characteristics for molecular subgroups shown in figure 3

| **General characteristics** | IDH-mut astrocytoma | IDH-mut oligodendroglioma |
| --- | --- | --- |
| Radiotherapy |  |  |
| n | 16 | 17 |
| Median age (years) | 34 | 46 |
| WHO grade II/III (%) | 44/56 | 47/53 |
| Resection (%) | 63 | 65 |
| Median TTF  *95%-CI* (years) | 2.0  *0.0-4.9* | 7.3  *3.7-9.6* |
| Median survival  *95%-CI* (years) | 12.1  *2.8-21.4* | n.r. |
| Temozolomide |  |  |
| n | 16 | 31 |
| Median age (years) | 34 | 46 |
| WHO grade II/III (%) | 62/38 | 35/65 |
| Resection (%) | 56 | 71 |
| Median TTF  *95%-CI* (years) | 2.3  *1.5-3.1* | 3.8  *2.6-4.9* |
| Median survival  *95%-CI* (years) | 6.1  *4.0-8.2* | 9.8  *6.7-12.9* |
| Radiotherapy + Temozolomide |  |  |
| n | 25 | 12 |
| Median age (years) | 38 | 47 |
| WHO grade II/III (%) | 36/64 | 25/75 |
| Resection (%) | 72 | 75 |
| Median TTF  *95%-CI* (years) | 6.6  *3.7-9.6* | n.r. |
| Median survival  *95%-CI* (years) | 8.4  *5.3-11.6* | n.r. |

*TTF* = time to treatment failure; *95%-CI* = 95% confidence interval; n.r. = not reached
