# Supplementary material for: A 25-year retrospective, single center analysis of 343 WHO grade II/III glioma patients: implications for grading and temozolomide therapy
Source: J Cancer Res Clin Oncol. 2021 Feb 4;147(8):2373–83. doi: 10.1007/s00432-021-03511-y (PMC8236451; doi:10.1007/s00432-021-03511-y)
Supplement: Supplementary file 7 — Supplementary file7 (DOCX 23 KB) [file 432_2021_3511_MOESM7_ESM.docx]

# Captions for supplementary figures

**Fig.S1** Identification of the study cohort

**Fig.S2** Changes when shifting from 2007 to 2016 WHO classification

Boxes at the top depict the histologic WHO 2007 classification of the whole cohort (n = 424, *A°II* = Astrocytoma WHO grade II, *A°III* = Astrocytoma WHO grade III, O*A°II* = Oligoastrocytoma WHO grade II, O*A°III* = Oligoastrocytoma WHO grade III, O*°II* = Oligodendroglioma WHO grade II, O*°III* = Oligodendroglioma WHO grade III). Boxes at the bottom depict the molecular groups based on the WHO 2016 classification (n = 257; 167 patients remained unclassified). The width of the boxes corresponds to the number of patients. Additional information on median follow-up in years (*f-u*) presenting symptoms (*Neurol. deficit* = neurologic deficit), tumor expansion, proportion of patients which underwent resection and distribution of the following first-line therapy (*RT* = radiotherapy, *CT* = chemotherapy, *W&S* = wait and see) is included in the boxes. Seizures were the dominating presenting symptom and more frequent in IDH-mutant than in IDH1-R132H-nm (IDH1-R132H-non-mutant) patients (64%/ 51%, p=0.04). While predominantly affecting the frontal lobe (70-75%) the average number of involved brain lobes was higher in IDH1-R132H-nm than in IDH-mutant tumors (1.7/ 1.4, p<0.01). The flows in between the upper and lower boxes show the redistribution of the patients, with the width being proportional to the patient number noted on the flow

**Fig.S3** OS impact of grading based on the 2007 WHO classification

Kaplan-Meier curves for overall survival (*OS*) for morphologic subgroups (WHO 2007, astrocytoma (a) and oligodendroglioma (b)). Differences between WHO grade II/III are only significant for astrocytoma (p< 0.05, log-ranked test)

**Fig.S4** Treatment allocation through three stages disease

Alluvial diagram with width of boxes and flows being proportional to the number of patients (individual numbers are noted within boxes or on flows). Boxes and flows are color coded depending on the kind of treatment. Note that the total number of patients shown at the top is decreasing for increasing therapy stages due to patient deaths or limitations in follow up. *RES* = proportion (%) of patients which underwent resection in addition to the treatment indicated by the block color; *FUT*= proportion (%) of patients with known follow-up treatment

**Fig.S5** Time to treatment failure for the first three therapy lines

Time to treatment failure (*TTF*) for all IDH-mutant tumors through first-line (black), second-line (red) and third-line (blue) therapy
